# 3-(4-Methyl­phen­yl)-5-phenyl-1,2-thia­zole

**DOI:** 10.1107/S2414314626008217

**Published:** 2026-08-20

**Authors:** A. S. Jeevan Chakravarthy, N. R. Sreenatha

**Affiliations:** a Department of Chemistry BMS Institute of Technology & Management Autonomous under Visvesvaraya Technological University, Avalahalli Yelahanka Bengaluru 560064 Karnataka India; b Research Center, Department of Physics, Government Engineering College, Bedarapura, Chamarajanagara 571313, Karnataka, India; Goethe-Universität Frankfurt, Germany

**Keywords:** single crystal XRD, monoclinic, iso­thia­zole, dihedral angles, crystal structure

## Abstract

The title compound, C_16_H_13_NS, crystallizes in the monoclinic space group *P*2_1_/*c* with two independent mol­ecules in the asymmetric unit. Both mol­ecules adopt a non-planar structure. No classical hydrogen bonds are found in the crystal structure.

## Abstract 2

The asymmetric unit of the title 3-(4-methyl­phen­yl)-5-phenyl-1,2-thia­zole compound comprises two independent mol­ecules with nearly planar geometries. No classical hydrogen bonds are found in the crystal structure.

## Graphical abstract

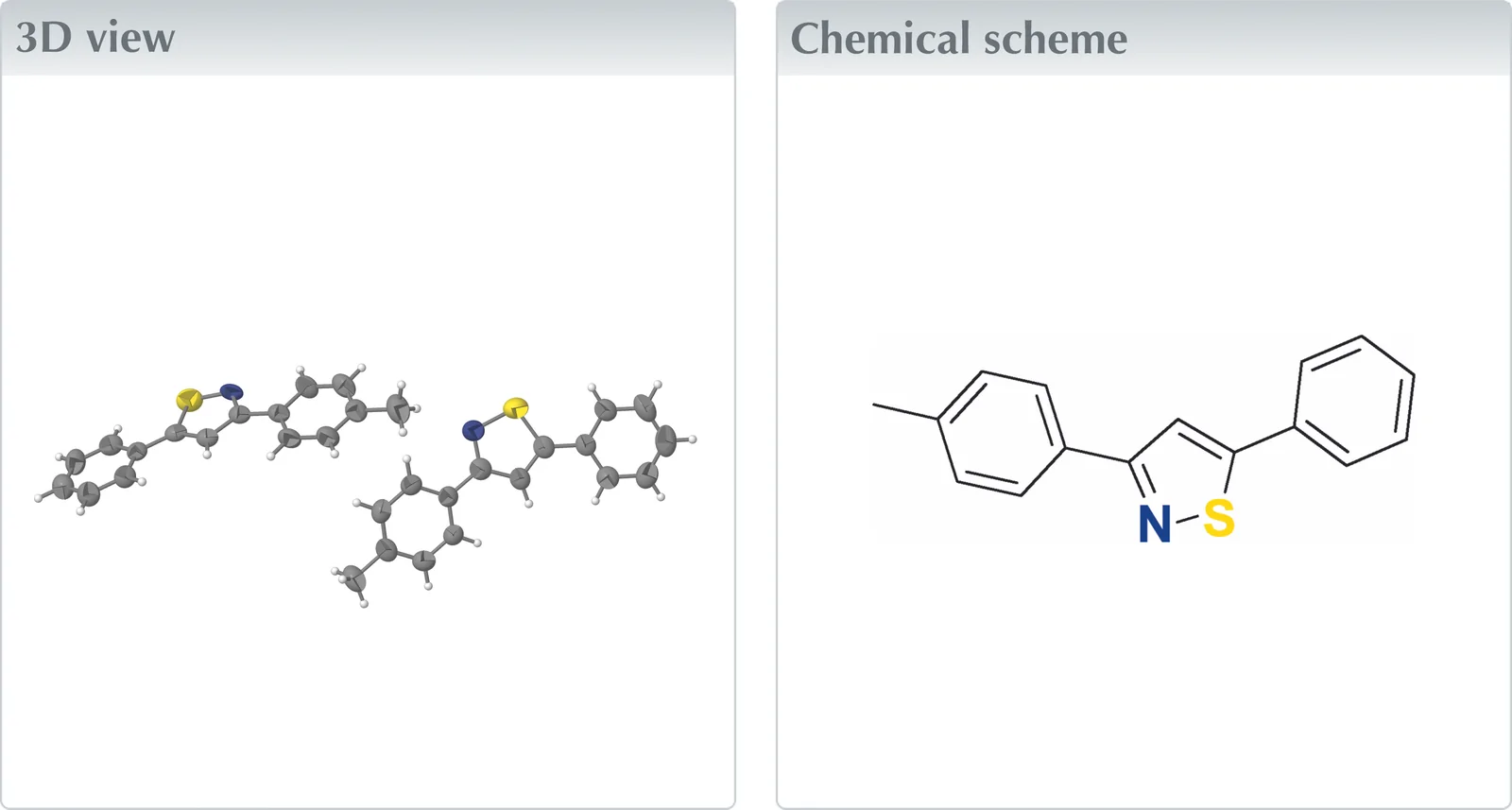

## Structure description

**Figure 1:**
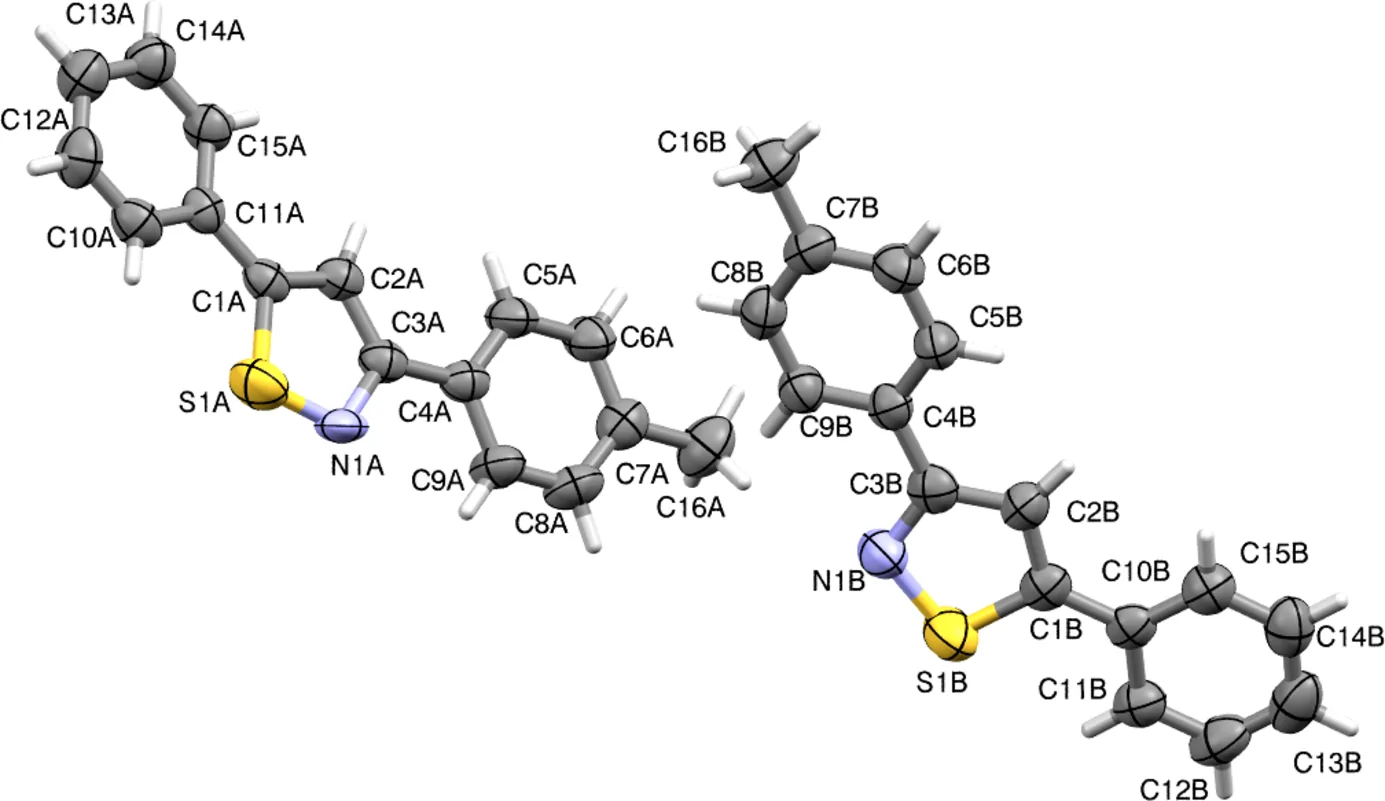
The mol­ecular structure of **2** with displacement ellipsoids drawn at the 50% probability level.

**Figure 2:**
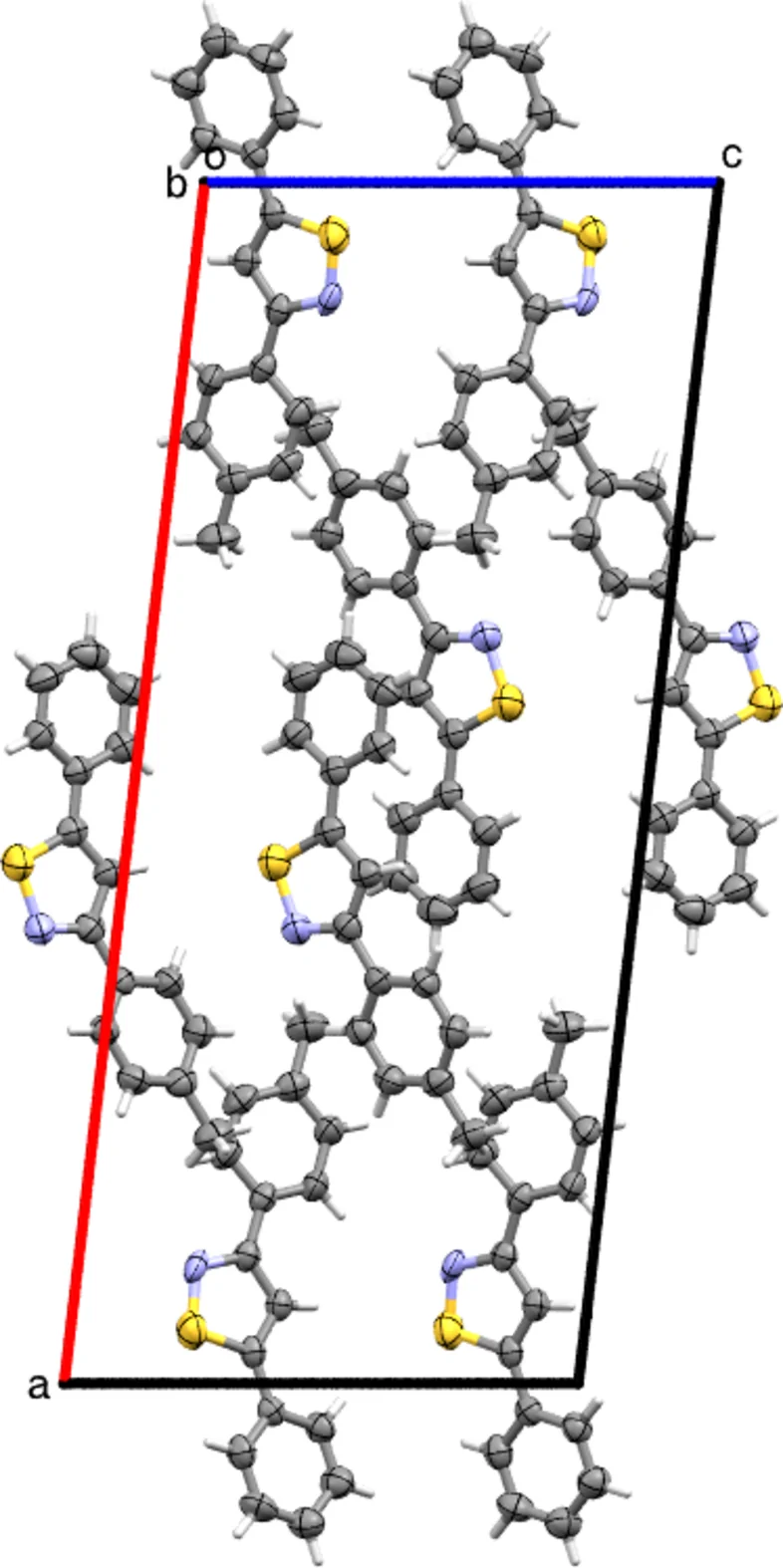
Packing of the title compound viewed along the *b* axis.

1,2-Thiazole derivatives are an important class of sulfur- and nitrogen-containing heterocyclic compounds and their structural diversity and electronic properties make them of interest in organic and medicinal chemistry (Kaberdin & Potkin, 2002 ▸). The asymmetric unit of the title compound (**2**) comprises two independent mol­ecules, *A* and *B* (Fig. 1 ▸). Both mol­ecules adopt a non-planar geometry as evidenced by the dihedral angles between the mean planes of the terminal phenyl rings (C4–C9 and C10–C15) of 11.1 (2) and 11.3 (2)° in molecules *A* and *B*, respectively. The bond lengths and bond angles are in good agreement with the literature values (Fait *et al.*, 2021 ▸; Guseinov *et al.*, 2026 ▸; Meundaeng *et al.*, 2019 ▸; Sreenatha *et al.*, 2018 ▸; Sreenatha *et al.*, 2021 ▸). There are no classical hydrogen bonds in the crystal structure.The packing of the title compound is shown in Fig. 2 ▸.

## Synthesis and crystallization

**Figure 3:**
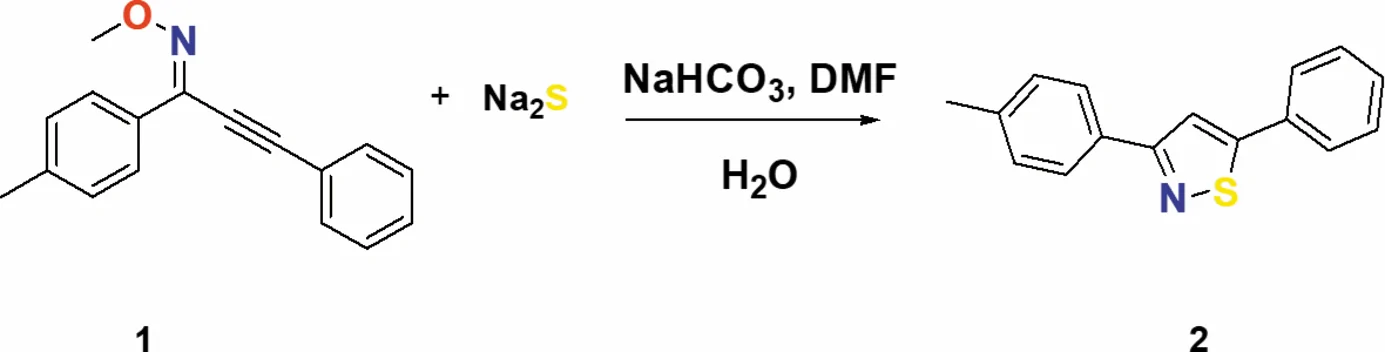
Synthesis scheme for the title compound.

The title compound was synthesized (Fig. 3 ▸) using a literature procedure (Zhang *et al.*, 2021 ▸). A pressure tube was charged with compound **1** [(*N*-methoxy-1-(4-methylphenyl)-3-phenylprop-2-yn-1-imine)] (0.2 mmol), Na_2_S (0.3 mmol, 23.4 mg), NaHCO_3_ (0.4 mmol, 33.6 mg), and H_2_O (1.2 mmol, 21.6 mg) in *N*,*N*-dimethylformamide (DMF) (2 ml) in the presence of air under sealed conditions.The reaction mixture was stirred at 90°C in an oil bath for 13–15 h until the starting material was consumed completely by TLC (spotted from micro work-up of aliquots). After the completion of the reaction, as indicated by the TLC, the mixture was poured into ethyl acetate and washed with brine (2 × 15 ml). The combined organic layers were dried over anhydrous MgSO_4_ and evaporated under vacuum. The residue was purified by flash column chromatography using petroleum ether/ethyl acetate as the eluent to afford the desired product **2** in 78% yield. 0.5 g of compound **2** were dissolved in 5 ml of DMF and allowed to undergo slow evaporation. Crystals obtained were isolated and subjected to single-crystal XRD studies.

## Refinement

Crystal data and structure refinement details are summarized in Table 1 ▸.

## Supplementary Material

Crystal structure: contains datablock(s) I. DOI: 10.1107/S2414314626008217/bt4204sup1.cif

Structure factors: contains datablock(s) I. DOI: 10.1107/S2414314626008217/bt4204Isup2.hkl

Supporting information file. DOI: 10.1107/S2414314626008217/bt4204Isup3.cml

CCDC reference: 2580089

Additional supporting information:  crystallographic information; 3D view; checkCIF report

## Figures and Tables

**Table 1:** Experimental details

Crystal data	
Chemical formula	C_16_H_13_NS
*M*_r_	251.33
Crystal system, space group	Monoclinic, *P*2_1_/*c*
Temperature (K)	293
*a*, *b*, *c* (Å)	28.7454 (18), 7.3669 (5), 12.2221 (7)
β (°)	96.675 (2)
*V* (Å^3^)	2570.7 (3)
*Z*	8
Radiation type	Mo *K*α
μ (mm^−1^)	0.23
Crystal size (mm)	0.27 × 0.25 × 0.22

Data collection	
Diffractometer	Bruker APEX
No. of measured, independent and observed [*I* > 2σ(*I*)] reflections	108451, 5931, 3157
*R*_int_	0.154
(sin θ/λ)_max_ (Å^−1^)	0.652

Refinement	
*R*[*F*^2^ > 2σ(*F*^2^)], *wR*(*F*^2^), *S*	0.078, 0.256, 1.03
No. of reflections	5931
No. of parameters	327
H-atom treatment	H-atom parameters constrained
Δρ_max_, Δρ_min_ (e Å^−3^)	0.55, −0.47

Computer programs: *APEX* (Bruker, 2012 ▸), *SAINT* (Bruker, 2012 ▸), *SHELXT 2018/2* (Sheldrick, 2015*a* ▸), *SHELXL2019/2* (Sheldrick, 2015*b* ▸) and *Mercury* (Macrae *et al.*, 2020 ▸).

## full crystallographic data

### 3-(4-Methylphenyl)-5-phenyl-1,2-thiazole Crystal data

**Table:**

C_16_H_13_NS	*F*(000) = 1056
*M**_r_* = 251.33	*D*_x_ = 1.299 Mg m^−^^3^
Monoclinic, *P*2_1_/*c*	Mo *K*α radiation, λ = 0.71073 Å
*a* = 28.7454 (18) Å	Cell parameters from 5931 reflections
*b* = 7.3669 (5) Å	θ = 2.9–27.6°
*c* = 12.2221 (7) Å	µ = 0.23 mm^−^^1^
β = 96.675 (2)°	*T* = 293 K
*V* = 2570.7 (3) Å^3^	Block, colorless
*Z* = 8	0.27 × 0.25 × 0.22 mm

### 3-(4-Methylphenyl)-5-phenyl-1,2-thiazole Data collection

**Table:**

Bruker APEX diffractometer	θ_max_ = 27.6°, θ_min_ = 2.9°
Radiation source: graphite	*h* = −37→37
*SAINT* (Bruker, 2012) scans	*k* = −9→9
108451 measured reflections	*l* = −15→15
5931 independent reflections	5931 standard reflections every 200 reflections
3157 reflections with *I* > 2σ(*I*)	intensity decay: none
*R*_int_ = 0.154	

### 3-(4-Methylphenyl)-5-phenyl-1,2-thiazole Refinement

**Table:**

Refinement on *F*^2^	0 restraints
Least-squares matrix: full	Hydrogen site location: inferred from neighbouring sites
*R*[*F*^2^ > 2σ(*F*^2^)] = 0.078	H-atom parameters constrained
*wR*(*F*^2^) = 0.256	*w* = 1/[σ^2^(*F*_o_^2^) + (0.1108*P*)^2^ + 2.3809*P*] where *P* = (*F*_o_^2^ + 2*F*_c_^2^)/3
*S* = 1.03	(Δ/σ)_max_ = 0.033
5931 reflections	Δρ_max_ = 0.55 e Å^−^^3^
327 parameters	Δρ_min_ = −0.47 e Å^−^^3^

### 3-(4-Methylphenyl)-5-phenyl-1,2-thiazole Special details

**Table:**

Geometry. All esds (except the esd in the dihedral angle between two l.s. planes) are estimated using the full covariance matrix. The cell esds are taken into account individually in the estimation of esds in distances, angles and torsion angles; correlations between esds in cell parameters are only used when they are defined by crystal symmetry. An approximate (isotropic) treatment of cell esds is used for estimating esds involving l.s. planes.
Refinement. The methyl H atoms were placed in calculated positions using the rotating-group model (HFIX 137) and refined as riding atoms with *U*(H)=1.5*U*_eq_(C). All other H atoms were positioned geometrically and refined using a riding model with *U*(H)=1.2*U*_eq_(C).

### 3-(4-Methylphenyl)-5-phenyl-1,2-thiazole Fractional atomic coordinates and isotropic or equivalent isotropic displacement parameters (Å^2^)

**Table:**

	*x*	*y*	*z*	*U*_iso_*/*U*_eq_	
S1A	0.04414 (5)	0.42640 (18)	0.76380 (9)	0.0778 (4)	
N1A	0.09737 (11)	0.3869 (5)	0.7700 (2)	0.0587 (9)	
C1A	0.02596 (13)	0.3317 (5)	0.6397 (3)	0.0492 (9)	
C2A	0.06513 (13)	0.2617 (5)	0.6003 (3)	0.0508 (9)	
C3A	0.10614 (13)	0.2910 (5)	0.6732 (3)	0.0508 (9)	
C4A	0.15425 (13)	0.2372 (5)	0.6552 (3)	0.0501 (9)	
C5A	0.16452 (14)	0.1602 (6)	0.5572 (3)	0.0607 (10)	
C6A	0.21023 (14)	0.1149 (6)	0.5426 (3)	0.0641 (11)	
C7A	0.24701 (14)	0.1462 (5)	0.6239 (3)	0.0608 (10)	
C8A	0.23581 (15)	0.2197 (6)	0.7218 (4)	0.0673 (11)	
C9A	0.19126 (14)	0.2632 (6)	0.7373 (3)	0.0626 (11)	
C10A	−0.02311 (12)	0.3329 (5)	0.5904 (3)	0.0475 (8)	
C11A	−0.05764 (15)	0.4254 (5)	0.6392 (3)	0.0600 (10)	
C12A	−0.10290 (15)	0.4257 (6)	0.5934 (4)	0.0705 (12)	
C13A	−0.11644 (15)	0.3342 (6)	0.4969 (4)	0.0702 (12)	
C14A	−0.08254 (15)	0.2414 (6)	0.4467 (4)	0.0667 (11)	
C15A	−0.03708 (14)	0.2408 (5)	0.4923 (3)	0.0563 (10)	
C16A	0.29642 (15)	0.0953 (7)	0.6074 (5)	0.0864 (15)	
H2A	0.064607	0.201873	0.533157	0.061*	
H5A	0.140541	0.138651	0.500818	0.073*	
H6A	0.216292	0.062322	0.476545	0.077*	
H8A	0.259638	0.239736	0.778802	0.081*	
H9A	0.185322	0.311734	0.804522	0.075*	
H11A	−0.049458	0.488321	0.704633	0.072*	
H12A	−0.125163	0.488810	0.627719	0.085*	
H13A	−0.147559	0.334402	0.465963	0.084*	
H14A	−0.091041	0.178990	0.381275	0.080*	
H15A	−0.014904	0.178016	0.457492	0.068*	
H16A	0.317120	0.193492	0.631155	0.130*	
H16B	0.298160	0.071144	0.530830	0.130*	
H16C	0.305499	−0.011301	0.649835	0.130*	
S1B	0.43349 (5)	0.5505 (2)	0.70884 (10)	0.0845 (5)	
N1B	0.37844 (12)	0.5644 (5)	0.6509 (3)	0.0685 (10)	
C1B	0.45945 (13)	0.6522 (5)	0.6095 (3)	0.0553 (10)	
C2B	0.42449 (13)	0.7000 (5)	0.5282 (3)	0.0572 (10)	
C3B	0.37930 (14)	0.6495 (5)	0.5530 (3)	0.0574 (10)	
C4B	0.33506 (13)	0.6768 (5)	0.4805 (3)	0.0524 (9)	
C5B	0.33329 (14)	0.7855 (6)	0.3882 (3)	0.0624 (11)	
C6B	0.29269 (15)	0.8109 (6)	0.3201 (3)	0.0686 (12)	
C7B	0.25081 (14)	0.7279 (6)	0.3411 (3)	0.0610 (10)	
C8B	0.25289 (14)	0.6182 (6)	0.4341 (4)	0.0642 (11)	
C9B	0.29366 (14)	0.5922 (6)	0.5025 (3)	0.0583 (10)	
C10B	0.50983 (13)	0.6700 (5)	0.6126 (3)	0.0523 (9)	
C11B	0.54025 (14)	0.5850 (6)	0.6950 (3)	0.0628 (11)	
C12B	0.58746 (16)	0.6008 (7)	0.6975 (4)	0.0801 (14)	
C13B	0.60644 (17)	0.7032 (7)	0.6184 (5)	0.0831 (15)	
C14B	0.57752 (16)	0.7858 (6)	0.5366 (4)	0.0732 (12)	
C15B	0.52998 (14)	0.7693 (6)	0.5326 (3)	0.0623 (10)	
C16B	0.20659 (15)	0.7537 (7)	0.2656 (4)	0.0795 (13)	
H2B	0.429909	0.759173	0.463708	0.069*	
H5B	0.360466	0.842966	0.371861	0.075*	
H6B	0.292833	0.884971	0.258554	0.082*	
H8B	0.225758	0.560620	0.450516	0.077*	
H9B	0.293740	0.517840	0.564046	0.070*	
H11B	0.527918	0.516673	0.748760	0.075*	
H12B	0.607110	0.542827	0.752498	0.096*	
H13B	0.638750	0.715640	0.621079	0.100*	
H14B	0.590270	0.853717	0.483222	0.088*	
H15B	0.510745	0.824993	0.475932	0.075*	
H16D	0.194645	0.637571	0.240169	0.119*	
H16E	0.212854	0.826209	0.203741	0.119*	
H16F	0.183835	0.813889	0.304443	0.119*	

### 3-(4-Methylphenyl)-5-phenyl-1,2-thiazole Atomic displacement parameters (Å^2^)

**Table:**

	*U*^11^	*U*^22^	*U*^33^	*U*^12^	*U*^13^	*U*^23^
S1A	0.0913 (9)	0.0871 (9)	0.0564 (6)	0.0087 (7)	0.0140 (6)	−0.0128 (6)
N1A	0.069 (2)	0.070 (2)	0.0332 (15)	−0.0157 (17)	−0.0129 (13)	0.0029 (14)
C1A	0.064 (2)	0.041 (2)	0.0427 (18)	−0.0008 (17)	0.0098 (16)	0.0043 (15)
C2A	0.061 (2)	0.047 (2)	0.0443 (19)	−0.0006 (18)	0.0054 (17)	0.0013 (16)
C3A	0.060 (2)	0.046 (2)	0.0458 (19)	−0.0005 (17)	0.0034 (16)	0.0044 (16)
C4A	0.059 (2)	0.043 (2)	0.0463 (19)	−0.0030 (17)	−0.0022 (16)	0.0026 (16)
C5A	0.063 (3)	0.074 (3)	0.043 (2)	0.002 (2)	−0.0046 (17)	−0.0007 (19)
C6A	0.060 (3)	0.080 (3)	0.051 (2)	0.012 (2)	0.0007 (18)	0.001 (2)
C7A	0.058 (2)	0.050 (2)	0.073 (3)	0.0021 (19)	0.001 (2)	0.006 (2)
C8A	0.064 (3)	0.062 (3)	0.069 (3)	−0.005 (2)	−0.016 (2)	−0.008 (2)
C9A	0.061 (3)	0.067 (3)	0.056 (2)	0.003 (2)	−0.0077 (19)	−0.013 (2)
C10A	0.054 (2)	0.040 (2)	0.0492 (19)	0.0006 (16)	0.0101 (16)	0.0062 (16)
C11A	0.074 (3)	0.054 (2)	0.054 (2)	0.001 (2)	0.0129 (19)	−0.0041 (18)
C12A	0.054 (3)	0.072 (3)	0.088 (3)	0.010 (2)	0.020 (2)	−0.003 (2)
C13A	0.060 (3)	0.070 (3)	0.080 (3)	−0.003 (2)	0.006 (2)	−0.005 (2)
C14A	0.065 (3)	0.065 (3)	0.070 (3)	−0.005 (2)	0.005 (2)	−0.009 (2)
C15A	0.059 (2)	0.054 (2)	0.057 (2)	0.0022 (19)	0.0120 (18)	−0.0084 (18)
C16A	0.061 (3)	0.085 (4)	0.110 (4)	0.008 (2)	0.000 (3)	−0.001 (3)
S1B	0.0821 (9)	0.1074 (11)	0.0650 (7)	0.0073 (7)	0.0128 (6)	0.0202 (7)
N1B	0.063 (2)	0.088 (3)	0.056 (2)	0.0101 (19)	0.0108 (16)	0.0065 (18)
C1B	0.057 (2)	0.047 (2)	0.061 (2)	0.0029 (17)	0.0046 (18)	−0.0113 (18)
C2B	0.053 (2)	0.057 (2)	0.061 (2)	0.0001 (18)	0.0033 (18)	−0.0039 (18)
C3B	0.062 (2)	0.054 (2)	0.057 (2)	0.0087 (19)	0.0096 (18)	−0.0096 (18)
C4B	0.051 (2)	0.057 (2)	0.051 (2)	0.0054 (18)	0.0099 (16)	−0.0064 (17)
C5B	0.055 (2)	0.073 (3)	0.059 (2)	−0.003 (2)	0.0104 (18)	0.009 (2)
C6B	0.070 (3)	0.077 (3)	0.059 (2)	0.003 (2)	0.007 (2)	0.010 (2)
C7B	0.057 (2)	0.060 (3)	0.065 (2)	0.008 (2)	0.0019 (19)	−0.011 (2)
C8B	0.054 (2)	0.069 (3)	0.072 (3)	−0.004 (2)	0.015 (2)	−0.003 (2)
C9B	0.060 (2)	0.060 (3)	0.056 (2)	−0.0007 (19)	0.0103 (18)	0.0038 (18)
C10B	0.054 (2)	0.047 (2)	0.055 (2)	0.0014 (17)	0.0033 (17)	−0.0122 (17)
C11B	0.061 (3)	0.065 (3)	0.061 (2)	0.004 (2)	0.0007 (19)	−0.004 (2)
C12B	0.057 (3)	0.092 (4)	0.087 (3)	0.013 (2)	−0.012 (2)	−0.017 (3)
C13B	0.059 (3)	0.086 (4)	0.106 (4)	−0.007 (3)	0.015 (3)	−0.037 (3)
C14B	0.069 (3)	0.068 (3)	0.085 (3)	−0.005 (2)	0.022 (2)	−0.010 (2)
C15B	0.064 (3)	0.056 (3)	0.067 (2)	0.002 (2)	0.005 (2)	−0.002 (2)
C16B	0.062 (3)	0.080 (3)	0.093 (3)	0.008 (2)	−0.009 (2)	−0.004 (3)

### 3-(4-Methylphenyl)-5-phenyl-1,2-thiazole Geometric parameters (Å, º)

**Table:**

S1A—N1A	1.551 (4)	S1B—N1B	1.660 (4)
S1A—C1A	1.696 (4)	S1B—C1B	1.674 (4)
C3A—C2A	1.409 (5)	C3B—N1B	1.353 (5)
C3A—N1A	1.425 (5)	C3B—C2B	1.417 (5)
C3A—C4A	1.479 (5)	C3B—C4B	1.477 (5)
C4A—C9A	1.388 (5)	C10B—C15B	1.399 (6)
C4A—C5A	1.388 (5)	C10B—C11B	1.403 (5)
C10A—C11A	1.394 (5)	C10B—C1B	1.450 (5)
C10A—C15A	1.395 (5)	C4B—C5B	1.380 (5)
C10A—C1A	1.468 (5)	C4B—C9B	1.397 (5)
C7A—C6A	1.383 (5)	C1B—C2B	1.374 (5)
C7A—C8A	1.386 (6)	C11B—C12B	1.359 (6)
C7A—C16A	1.505 (6)	C11B—H11B	0.9300
C1A—C2A	1.375 (5)	C9B—C8B	1.371 (6)
C2A—H2A	0.9300	C9B—H9B	0.9300
C11A—C12A	1.355 (6)	C7B—C8B	1.390 (6)
C11A—H11A	0.9300	C7B—C6B	1.400 (6)
C8A—C9A	1.354 (6)	C7B—C16B	1.494 (6)
C8A—H8A	0.9300	C5B—C6B	1.365 (5)
C9A—H9A	0.9300	C5B—H5B	0.9300
C5A—C6A	1.387 (5)	C2B—H2B	0.9300
C5A—H5A	0.9300	C8B—H8B	0.9300
C12A—C13A	1.374 (6)	C6B—H6B	0.9300
C12A—H12A	0.9300	C15B—C14B	1.367 (6)
C15A—C14A	1.360 (5)	C15B—H15B	0.9300
C15A—H15A	0.9300	C12B—C13B	1.387 (7)
C6A—H6A	0.9300	C12B—H12B	0.9300
C14A—C13A	1.390 (6)	C14B—C13B	1.367 (7)
C14A—H14A	0.9300	C14B—H14B	0.9300
C13A—H13A	0.9300	C13B—H13B	0.9300
C16A—H16A	0.9600	C16B—H16D	0.9600
C16A—H16B	0.9600	C16B—H16E	0.9600
C16A—H16C	0.9600	C16B—H16F	0.9600
			
N1A—S1A—C1A	99.56 (17)	N1B—S1B—C1B	98.40 (18)
C2A—C3A—N1A	112.6 (3)	N1B—C3B—C2B	115.0 (4)
C2A—C3A—C4A	126.4 (3)	N1B—C3B—C4B	119.4 (4)
N1A—C3A—C4A	121.0 (3)	C2B—C3B—C4B	125.6 (4)
C9A—C4A—C5A	117.3 (4)	C15B—C10B—C11B	117.5 (4)
C9A—C4A—C3A	120.5 (3)	C15B—C10B—C1B	121.5 (4)
C5A—C4A—C3A	122.3 (3)	C11B—C10B—C1B	121.0 (4)
C11A—C10A—C15A	117.2 (3)	C5B—C4B—C9B	117.8 (4)
C11A—C10A—C1A	121.7 (3)	C5B—C4B—C3B	121.1 (4)
C15A—C10A—C1A	121.1 (3)	C9B—C4B—C3B	121.1 (4)
C6A—C7A—C8A	116.8 (4)	C3B—N1B—S1B	106.9 (3)
C6A—C7A—C16A	121.1 (4)	C2B—C1B—C10B	129.8 (4)
C8A—C7A—C16A	122.0 (4)	C2B—C1B—S1B	106.8 (3)
C2A—C1A—C10A	130.2 (3)	C10B—C1B—S1B	123.3 (3)
C2A—C1A—S1A	106.7 (3)	C12B—C11B—C10B	121.0 (4)
C10A—C1A—S1A	123.1 (3)	C12B—C11B—H11B	119.5
C1A—C2A—C3A	112.3 (3)	C10B—C11B—H11B	119.5
C1A—C2A—H2A	123.8	C8B—C9B—C4B	120.4 (4)
C3A—C2A—H2A	123.8	C8B—C9B—H9B	119.8
C3A—N1A—S1A	108.7 (2)	C4B—C9B—H9B	119.8
C12A—C11A—C10A	121.4 (4)	C8B—C7B—C6B	116.6 (4)
C12A—C11A—H11A	119.3	C8B—C7B—C16B	122.1 (4)
C10A—C11A—H11A	119.3	C6B—C7B—C16B	121.3 (4)
C9A—C8A—C7A	122.1 (4)	C6B—C5B—C4B	121.7 (4)
C9A—C8A—H8A	119.0	C6B—C5B—H5B	119.2
C7A—C8A—H8A	119.0	C4B—C5B—H5B	119.2
C8A—C9A—C4A	121.6 (4)	C1B—C2B—C3B	112.9 (4)
C8A—C9A—H9A	119.2	C1B—C2B—H2B	123.5
C4A—C9A—H9A	119.2	C3B—C2B—H2B	123.5
C6A—C5A—C4A	120.6 (4)	C9B—C8B—C7B	122.2 (4)
C6A—C5A—H5A	119.7	C9B—C8B—H8B	118.9
C4A—C5A—H5A	119.7	C7B—C8B—H8B	118.9
C11A—C12A—C13A	121.1 (4)	C5B—C6B—C7B	121.4 (4)
C11A—C12A—H12A	119.4	C5B—C6B—H6B	119.3
C13A—C12A—H12A	119.4	C7B—C6B—H6B	119.3
C14A—C15A—C10A	121.2 (4)	C14B—C15B—C10B	121.0 (4)
C14A—C15A—H15A	119.4	C14B—C15B—H15B	119.5
C10A—C15A—H15A	119.4	C10B—C15B—H15B	119.5
C7A—C6A—C5A	121.6 (4)	C11B—C12B—C13B	120.2 (5)
C7A—C6A—H6A	119.2	C11B—C12B—H12B	119.9
C5A—C6A—H6A	119.2	C13B—C12B—H12B	119.9
C15A—C14A—C13A	120.6 (4)	C13B—C14B—C15B	120.4 (5)
C15A—C14A—H14A	119.7	C13B—C14B—H14B	119.8
C13A—C14A—H14A	119.7	C15B—C14B—H14B	119.8
C12A—C13A—C14A	118.5 (4)	C14B—C13B—C12B	119.8 (5)
C12A—C13A—H13A	120.8	C14B—C13B—H13B	120.1
C14A—C13A—H13A	120.8	C12B—C13B—H13B	120.1
C7A—C16A—H16A	109.5	C7B—C16B—H16D	109.5
C7A—C16A—H16B	109.5	C7B—C16B—H16E	109.5
H16A—C16A—H16B	109.5	H16D—C16B—H16E	109.5
C7A—C16A—H16C	109.5	C7B—C16B—H16F	109.5
H16A—C16A—H16C	109.5	H16D—C16B—H16F	109.5
H16B—C16A—H16C	109.5	H16E—C16B—H16F	109.5
			
C2A—C3A—C4A—C9A	−175.5 (4)	N1B—C3B—C4B—C5B	170.4 (4)
N1A—C3A—C4A—C9A	6.4 (6)	C2B—C3B—C4B—C5B	−11.9 (6)
C2A—C3A—C4A—C5A	4.7 (6)	N1B—C3B—C4B—C9B	−10.1 (6)
N1A—C3A—C4A—C5A	−173.4 (3)	C2B—C3B—C4B—C9B	167.6 (4)
C11A—C10A—C1A—C2A	−174.2 (4)	C2B—C3B—N1B—S1B	0.2 (4)
C15A—C10A—C1A—C2A	5.7 (6)	C4B—C3B—N1B—S1B	178.2 (3)
C11A—C10A—C1A—S1A	5.2 (5)	C1B—S1B—N1B—C3B	−0.2 (3)
C15A—C10A—C1A—S1A	−174.8 (3)	C15B—C10B—C1B—C2B	9.9 (6)
N1A—S1A—C1A—C2A	0.6 (3)	C11B—C10B—C1B—C2B	−168.9 (4)
N1A—S1A—C1A—C10A	−179.0 (3)	C15B—C10B—C1B—S1B	−174.5 (3)
C10A—C1A—C2A—C3A	179.3 (3)	C11B—C10B—C1B—S1B	6.7 (5)
S1A—C1A—C2A—C3A	−0.2 (4)	N1B—S1B—C1B—C2B	0.2 (3)
N1A—C3A—C2A—C1A	−0.3 (4)	N1B—S1B—C1B—C10B	−176.2 (3)
C4A—C3A—C2A—C1A	−178.5 (3)	C15B—C10B—C11B—C12B	0.8 (6)
C2A—C3A—N1A—S1A	0.7 (4)	C1B—C10B—C11B—C12B	179.7 (4)
C4A—C3A—N1A—S1A	179.0 (3)	C5B—C4B—C9B—C8B	−0.2 (6)
C1A—S1A—N1A—C3A	−0.7 (3)	C3B—C4B—C9B—C8B	−179.7 (4)
C15A—C10A—C11A—C12A	0.1 (6)	C9B—C4B—C5B—C6B	0.1 (6)
C1A—C10A—C11A—C12A	−179.9 (4)	C3B—C4B—C5B—C6B	179.6 (4)
C6A—C7A—C8A—C9A	−1.5 (7)	C10B—C1B—C2B—C3B	176.0 (4)
C16A—C7A—C8A—C9A	−179.1 (4)	S1B—C1B—C2B—C3B	−0.2 (4)
C7A—C8A—C9A—C4A	−0.3 (7)	N1B—C3B—C2B—C1B	0.0 (5)
C5A—C4A—C9A—C8A	1.6 (6)	C4B—C3B—C2B—C1B	−177.8 (3)
C3A—C4A—C9A—C8A	−178.2 (4)	C4B—C9B—C8B—C7B	0.0 (6)
C9A—C4A—C5A—C6A	−1.2 (6)	C6B—C7B—C8B—C9B	0.2 (6)
C3A—C4A—C5A—C6A	178.7 (4)	C16B—C7B—C8B—C9B	179.0 (4)
C10A—C11A—C12A—C13A	0.1 (7)	C4B—C5B—C6B—C7B	0.1 (7)
C11A—C10A—C15A—C14A	−0.2 (6)	C8B—C7B—C6B—C5B	−0.3 (6)
C1A—C10A—C15A—C14A	179.8 (4)	C16B—C7B—C6B—C5B	−179.1 (4)
C8A—C7A—C6A—C5A	1.9 (6)	C11B—C10B—C15B—C14B	−1.5 (6)
C16A—C7A—C6A—C5A	179.5 (4)	C1B—C10B—C15B—C14B	179.7 (4)
C4A—C5A—C6A—C7A	−0.6 (7)	C10B—C11B—C12B—C13B	0.5 (7)
C10A—C15A—C14A—C13A	0.0 (6)	C10B—C15B—C14B—C13B	0.8 (7)
C11A—C12A—C13A—C14A	−0.3 (7)	C15B—C14B—C13B—C12B	0.5 (7)
C15A—C14A—C13A—C12A	0.2 (7)	C11B—C12B—C13B—C14B	−1.1 (7)
